# US local public health department spending between 2008 and 2016 did not increase for communities in need

**DOI:** 10.1186/s12913-022-07613-2

**Published:** 2022-02-21

**Authors:** Olivia Reszczynski, John Connolly, Kaitlyn Shultz, Sheila Kelly, Nandita Mitra, Jeffrey Hom, Atheendar Venkataramani, Krisda H. Chaiyachati

**Affiliations:** 1 Medical University of South Carolina, Charleston, SC USA; 2 The Perelman School of Medicine at the University of Pennsylvania, Philadelphia, PA USA; 3 Department of Biostatistics, Epidemiology, & Informatics, University of Pennsylvania, Philadelphia, USA; 4 Philadelphia Department of Public Health, Philadelphia, USA

**Keywords:** Public health, Healthcare policy, Community health

## Abstract

**Background:**

Greater US local public health department (LPHD) spending has been associated with decreases in population-wide mortality. We examined the association between changes in LPHD spending between 2008 and 2016 and county-level sociodemographic indicators of public health need.

**Methods:**

Multivariable linear regression was used to estimate the association between changes in county-level per-capita LPHD spending and 2008 sociodemographic indicators of interest: percent of population that was over 65 years old, Black, Hispanic, in poverty, unemployed, and uninsured. A second model assessed the relationship between changes in LPHD spending and sociodemographic shifts between 2008 and 2016.

**Results:**

LPHD spending increases were associated with higher percentage points of 2008 adults over 65 years of age (+$0.53 per higher percentage point; 95% CI: +$0.01 to +$1.06) and unemployment (−$1.31; 95% CI: −$2.34 to −$0.27). Spending did not increase for communities with a higher proportion of people who identified as Black or Hispanic, or those with a greater proportion of people in poverty or uninsured, using either baseline or sociodemographic shifts between 2008 and 2016.

**Conclusion:**

Future LPHD funding decisions should consider increasing investments in counties serving disadvantaged communities to counteract the social, political, and structural barriers which have historically prevented these communities from achieving better health.

**Supplementary Information:**

The online version contains supplementary material available at 10.1186/s12913-022-07613-2.

## Background

The evidence supporting the value of US local public health departments (LPHDs), who financially support infrastructure or programs designed to promote health and prevent disease and injury, has been consistently positive. Since the early 2000’s, increases in LPHD spending have been associated with decreases in population-wide mortality from preventable conditions such as influenza and cardiovascular disease [1–4]. For example, in California between 2001 and 2009, each $10 per capita increase in public health spending was associated with all-cause mortality declines of 9.1 per 100,000 deaths [4]. Yet, since 2001, spending on LPHDs has dropped by 18%, and at least 50,000 state and local public health jobs have been eliminated [5, 6]. Relative to the annual $3.6 trillion that the US spends on health care, total public health spending is 3% [7, 8].

In the past decade, the US has faced a number of public health crises - the opioid epidemic, renewed outbreaks of HIV, and the COVID-19 pandemic [5–8]. These crises have disproportionately affected older adults, racial and ethnic minorities, and the impoverished [9]. Given these dynamics, it is imperative to examine how public health investments in disadvantaged counties have changed over time. Such knowledge will illuminate how federal, state, and local policymakers should consider plans for allocating or reallocating funds to LPHDs to localities that have experienced disproportionally worse health and public health crises, addressing ongoing public health crises in the near term, and preventing those that may arise in the future.

Public health funding in the United States is comprised of a mix of federal, state, and local dollars, and spending in a given geographic area is an aggregation of spending from these sources [6, 9, 10]. Approximately 27% of funding for LPHDs is supported by federal sources, with a greater proportion of funding derived from state tax revenues, local tax revenues, and grants [5]. The absolute quantity and proportion of non-federal dollars supporting LPHDs varies within and across states, and whether this variation reflects the variable needs of local communities is understudied.

To examine investments in LPHDs relative to the demographic makeup of their county, we used LPHD spending as a proxy for local investments in public health and conducted a county-level cohort study to estimate the association between changes in LPHD spending between 2008 and 2016 and (a) baseline 2008 sociodemographics in the LPHD’s county and (b) sociodemographics shifts in the LPHD’s county between 2008 and 2016.

## Methods

We measured public health department spending using 2008 and 2016 data from a survey of LPHD’s conducted regularly by the National Association of County and City Health Officials’ National Profile of Local Health Departments [10].

County-level 2008 and 2016 sociodemographics were obtained from the following sources: (a) the American Community Survey for age, racial, and ethnic distributions; (b) the US Census Bureau’s Small Area Income and Poverty Estimates for poverty data; (c) the Bureau of Labor & Statistics for unemployment data; (d) the Small Area Health Insurance Estimates to measure the rates of uninsured; and (e) the National Center for Health Statistics’ determination of a county’s rurality [11–13]. We excluded counties that received funds from more than one LPHD and excluded LPHDs that spent funds in more than one county to isolate a one-to-one relationship between LPHD spending and county demographics. Our final cohort consisted of 793 (26%) out of 3006 counties in the US.

To standardize spending, each LPHD’s expenditure was divided by the total number of adults in their county to calculate per capita LPHD spending in 2008 and 2016. Per capita spending in 2008 was inflation-adjusted to match 2016 dollars using the Consumer Price Index. Changes in per capita spending, the primary outcome, was the difference between 2016 and 2008 per-capita spending.

Multivariable linear regression was used to estimate the association between changes in per capita spending in each county and 2008 sociodemographic measures of interest: the percent of the population that was over 65 years old, Black, Hispanic, in poverty, unemployed, and uninsured (Additional file 1: Table 1). A separate multivariable linear model was used to investigate the association between changes in per capita spending and shifts in sociodemographics between 2008 and 2016. All models adjusted for LPHD’s baseline 2008 per capita spending and a county’s rurality, the median household income in 2008, and shifts in the median income during the study period. Analyses were conducted using SAS version 9.4 (SAS Institute Inc).

## Results

Counties included in our analytic cohort tended to be younger, wealthier, have fewer racial and ethnic minorities, and have fewer uninsured relative to the national average of all counties (Table 1, Additional file 2: Table 2).

**Table 1:** County Characteristics and the Adjusted Association Between 2008 to 2016 Per Capita Local Public Health Department Spending and Percentage Point Differences in County-level (a) 2008 Sociodemographics and (b) 2008 to 2016 Sociodemographic Shifts

	County-level demographics		Changes in per capita spending	
Sociodemographic characteristics	Characteristics of all US counties in 2008 (*N* = 3005), mean (SD)	Characteristics of sampled counties in 2008 (*N* = 793), mean (SD)	Based on 2008 sociodemographics^a^ (95% CI)	Based on 2008 to 2016 sociodemographic shifts^b^ (95% CI)
% over 65 years old	16.1 (4.2)	15.8 (4.2)	+ $0.53* (+ $0.01 to + $1.06)	− $2.79** (− $4.18 to − $1.40)
% Black	8.3 (14.0)	7.3 (12.1)	+ $0.00 (− $0.16 to + $0.17)	− $0.40 (− $2.39 to + $1.58)
% Hispanic	8.5 (13.5)	7.2 (10.3)	+ $0.02 (− $0.19 to + $0.22)	+ $0.56 (− $1.09 to + $2.21)
% in poverty	15.1 (6.0)	13.9 (5.2)	− $0.39 (− $1.10 to + $0.32)	− $0.08 (− $1.20 to + $1.04)
% unemployed	5.8 (2.1)	6.0 (1.8)	− $1.31* (− $2.34 to − $0.27)	− $0.01 (− $1.26 to + $1.25)
% uninsured	14.3 (4.7)	12.9 (4.0)	− $0.14 (− $0.70 to + $0.41)	− $0.55 (− $1.20 to + $0.10)

^a^All reported values were from the same linear model, which included each of the 2008 county-level sociodemographic characteristics (percent over 65 years old, Black, Hispanic, in poverty, unemployed, and uninsured) and were additionally adjusted for baseline per capita spending in 2008, the median household income within a county, and a binary determination rurality

^b^All reported values were from the same linear model, which included 2008 to 2016 shifts in county-level sociodemographic characteristics (percent over 65 years old, Black, Hispanic, in poverty, unemployed, and uninsured) and were additionally adjusted for baseline per capita spending in 2008, shifts in the median household income within a county from 2008 to 2016, and a binary determination of rurality

*p < 0.05; **p < 0.001

County-level, 2008 sociodemographic characteristics associated with changes in per capita LPHD spending included age and the unemployment rate (Table 1). For every additional percentage point in the proportion of a county over 65 years old, per capita spending increased by an estimated +$0.53 (95% CI: +$0.01 to +$1.06) and for every additional percentage point in the proportion of the population who was unemployed, per capita spending decreased by −$1.31 (95% CI: −$2.34 to −$0.27).

When evaluating the association between 2008 to 2016 demographic shifts with changes in per capita LPHD spending, only an increase in the proportion of the population that was 65 years or older was statistically significant. For every additional percentage point, per capita spending decreased by −$2.79 (95% CI: −$4.18 to −$1.40).

## Discussion

Sociodemographic characteristics were weakly associated with LPHD’s per capita changes in spending. The proportion of older adults was associated with increased per capita spending in counties with an older population in 2008 but decreased in counties with an increased proportion of older adults by 2016. The unemployment rate in 2008 was associated with less per capita LPHD spending by 2016. These findings likely reflect shifts in LPHD’s local tax-base, a major revenue stream, due to a greater number of retirees and unemployed.

More importantly, our results are noteworthy for what we did not find. Spending did not increase for communities with a higher proportion of people who identified as Black or Hispanic, or those with a greater proportion of poverty or lack of health insurance. These are markers of communities who have faced historic and structural barriers impeding their health and well-being, reflected by trends in higher overall mortality and, more recently, a greater burden of disease and public health crises, such as the COVID-19 and the opioid epidemic [8, 9]. To correct these enduring trends, greater financial investments will need to be targeted to disadvantaged communities to address ongoing public health crises in the near term and preventing those that may arise in the future. At the federal level, where spending is not dependent on fluctuations in local tax revenue, allocations to LPHDs could account for local needs by using sociodemographic measures including age, unemployment rate, or the social vulnerability index [14].

This study has limitations. Our study is cross sectional and therefore does not support causal conclusions. Because not all counties have a LPHD and many LPHD support multiple counties, the generalizability of our findings is limited to counties that are served by only one LPHD. We could not accurately account for state and federal public health investments within each county if not provided through their LPHD. We were not able to capture what programs LPHDs were able to support or required to support, such as tobacco or opioid prevention programs, due to requirements from state or federal funding sources. Finally, we also could not capture whether LPHD concentrated their spending in disadvantaged communities within each county or focused public health programming on conditions that disproportionately impacted disadvantaged populations.

## Conclusions

In total, while the US spends $3.6 trillion dollars annually on health care, only 3% of that is spent on public health. This funding distribution does not reflect the vital role local public health departments have in managing the public health crises we face today and preventing them in the future. This study found that public health department spending did not increase for communities with a higher proportion of racial and ethnic minorities, or those with a greater proportion of poverty or lack of health insurance. Because public health crises disproportionately affect disadvantaged populations such as older adults, racial and ethnic minorities, and the poor, mechanisms to increase LPHD funding from the federal, state, and local level should all be urgently explored. Targeted funding to communities with higher proportions of traditionally disadvantaged populations will be one important step to counteract the social, political, and structural barriers which have historically prevented these groups from achieving better health.

## Supplementary Information

**Additional file 1: Table 1.** Definitions of Sociodemographic Measures Related Unemployment, Uninsured, and Poverty.

**Additional file 2: Table 2.** 2008 Characteristics of Local Public Health Departments’ Counties in the Analytic Cohort and Across the US.

## Data Availability

Data and materials are available by emailing Dr. Krisda H. Chaiyachati at Krisda.Chaiyachati@pennmedicine.upenn.edu. Data was obtained from obtained from the following sources: (a) the American Community Survey for age, racial, and ethnic distributions; (b) the US Census Bureau’s Small Area Income and Poverty Estimates for poverty data; (c) the Bureau of Labor & Statistics for unemployment data; (d) the Small Area Health Insurance Estimates to measure the rates of uninsured; and (e) the National Center for Health Statistics’ determination of a county’s rurality.

## Abbreviation

LPHD: Local public health department
