# Supplementary material for: US local public health department spending between 2008 and 2016 did not increase for communities in need
Source: BMC Health Serv Res. 2022 Feb 21;22:237. doi: 10.1186/s12913-022-07613-2 (PMC8860251; doi:10.1186/s12913-022-07613-2)
Supplement: Supplementary file 1 — Additional file 1: Table 1. Definitions of Sociodemographic Measures Related Unemployment, Uninsured, and Poverty. [file 12913_2022_7613_MOESM1_ESM.docx]

| **Sociodemographic Term** | **Definition and Source** |
| --- | --- |
| Percent unemployed | Statistic taken from the U.S. Bureau of Labor Statistics, calculated as the percentage of the labor force that does not have a job, is looking for a job, and is available for work. The labor force includes people that are employed and unemployed only.^12^ |
| Percent uninsured | Statistic taken from the U.S Census Bureau Small Area Health Insurance Estimates (SAHIE), calculated as the estimated number of uninsured individuals under the age of 65. The model uses data from the American Community Survey (ACS) with Medicaid enrollment data and additional census data.^13^ |
| Percent in poverty | Statistic taken from the U.S Census Bureau Small Area Income and Poverty Estimates (SAIPE), calculated as the percent of people of all ages living in poverty. This includes children under the age of 5, children 5 to 17 years of age, and children under the age of 18. The model uses data from ACS with additional information from federal tax records and other sources^14^ |
