# Supplementary material for: US local public health department spending between 2008 and 2016 did not increase for communities in need
Source: BMC Health Serv Res. 2022 Feb 21;22:237. doi: 10.1186/s12913-022-07613-2 (PMC8860251; doi:10.1186/s12913-022-07613-2)
Supplement: Supplementary file 2 — Additional file 2: Table 2. 2008 Characteristics of Local Public Health Departments’ Counties in the Analytic Cohort and Across the US. [file 12913_2022_7613_MOESM2_ESM.docx]

| **2008 Characteristics** | **Counties in Cohort** | **All Counties in the US** |
| --- | --- | --- |
|  | **N=793** | **N=3005** |
|  |  |  |
| **Population served, n** | **120,069,662** | **127,430,663** |
|  |  |  |
| **Region, n (%)** |  |  |
| Northeast | 60 (7.6) | 298 (9.9) |
| Southeast | 232 (29.3) | 906 (30.2) |
| Midwest | 326 (41.1) | 855 (28.5) |
| West | 175 (22.1) | 946 (31.5) |
